# Design of Polypropylene Electret Melt Blown Nonwovens with Superior Filtration Efficiency Stability through Thermally Stimulated Charging

**DOI:** 10.3390/polym12102341

**Published:** 2020-10-13

**Authors:** Haifeng Zhang, Nuo Liu, Qianru Zeng, Jinxin Liu, Xing Zhang, Mingzheng Ge, Wei Zhang, Suying Li, Yijun Fu, Yu Zhang

**Affiliations:** 1 College of Textile and Clothing, Nantong University, Nantong 226019, China; zhanghf@ntu.edu.cn (H.Z.); ln63kk@outlook.com (N.L.); zeng990504@163.com (Q.Z.); mzge1990@ntu.edu.cn (M.G.); lsy@ntu.edu.cn (S.L.); fuyj@ntu.edu.cn (Y.F.); z.yu@ntu.edu.cn (Y.Z.); 2 National & Local Joint Engineering Research Center of Technical Fiber Composites for Safety and Health, College of Textile and Clothing, Nantong University, Nantong 226019, China; 3 Engineering Research Center of Technical Textiles, Ministry of Education, Donghua University, Shanghai 201620, China; drliujx@163.com (J.L.); tulip_90@163.com (X.Z.)

**Keywords:** melt blown, electret, thermally stimulated, annealing, thermal charging

## Abstract

Electret filters are widely used in particulate matter filtration due to their filtration efficiency that can be greatly improved by electrostatic forces without sacrificing the air resistance. However, the attenuation of the filtration efficiency remains a challenge. In this study, we report a novel strategy for producing an electret melt blown filter with superior filtration efficiency stability through a thermally stimulated charging method. The proposed approach optimizes the crystal structure and therefore results in the increased production probability of the charge traps. In addition, the re-trapping phenomenon caused by the thermal stimulation during the charging process can greatly increase the proportion of deep charge to shallow charge and improve the charge stability. A superior electret melt blown filtration material with a high filtration efficiency of 99.65%, low pressure drop of 120 Pa, and satisfactory filtration efficiency stability was produced after three cyclic charging times. The excellent filtration performance indicated that the developed material is a good air filtration candidate component for personal protection applications.

## 1. Introduction

The coronavirus disease 2019 (COVID-19) has caused major disruptions to nearly all facets of daily life around the world [1]. This disease is transmitted through aerosol droplets. However, an effective method for getting rid of COVID-19 is not yet developed. The current preventive approaches include wearing personal protective masks, such as N95 respirators. In addition to the disease, fine particulate matter (PM) pollution has drawn worldwide attention due to its serious threat to public health, daily lives, visibility, climate, and ecosystems [2,3,4]. Electret filters are widely used in PM filtration and virus defense due to their cost-effectiveness, energy-saving features, high filtration efficiency, and low pressure drop [5]. The electret melt blown nonwoven is a dominantly used material for respirators due to its fine diameter, small pore size, high porosity, 3D porous network structure, and quasi-permanent electrostatic interaction [6]. The electret melt blown nonwoven used as an air filter has a special filtering electrostatic mechanism besides normally inertial capture, interception, gravitational capture, and diffusion mechanisms. Benefiting from the ability of quasi-permanent reserving of abundant charges and creating an external macroscopic electric field on the periphery of fibers, the COVID-19 virus with a size of about 100 nm can also be captured by the electrostatic attraction. Additionally, compared with those of other traditional filtration materials, the filtration efficiency of electret melt blown nonwoven can be significantly improved without increasing the pressure drop. Therefore, this material is widely used in surgical face masks; air-purifying respirators; heating ventilation air conditioning (HVAC) filters; and automotive cabin air filters [7].

The most noteworthy performances of electret melt blown filters are the amount and duration of the implanted charges, which can determine the strength of electrostatic attraction. To date, there are mainly two ways to improve electret performance. One approach is to blend additives with a polymer during the melt blown process, which can optimize the crystal structure of the material and provide more traps for the charge storage. Another approach is to improve the charging technology and optimize the charging parameters. Electret filters can be produced using various methods, including corona, triboelectric, induction, and water jet charging. Among these methods, corona charging is commonly used because it is suitable for charging all kinds of fibers [8]. Corona is a sustainable and non-disruptive electrical discharge method between asymmetric electrodes that can be used when a sufficiently high potential difference exists [9]. In the corona discharge method, the key process factors are known to be applied voltage, charging time, charging distance, and charging temperature and humidity. Several researchers investigated the influences of these factors on the surface voltage [10]. For example, Thakur et al. observed that the applied voltage and charging time had a positive effect on the surface voltage of the electret material, whereas the charging distance exhibits negative effects [8]. Chen et al. found that a threshold for applied voltage exists, and this threshold depends on the polarity of charging [11] Gao et al. investigated the effects of charging temperature and charging time on the surface potential decay of the electret materials, and found that charge stability could be promoted by means of thermal treatment with proper parameters of charging temperature and charging time [12]. Nevertheless, these studies focused on the relationships between the filtration efficiency and the process factors; the stability of the filtration efficiency stability was rarely examined.

The charge of electret materials will decay after a certain period of time, and the charge storage stability hampers the filtration performance of the electret filters to a great extent [13]. To improve the charge stability, several researchers attempted to charge the material at thermal conditions. Lin et al. discovered that the surface voltage of polypropylene nonwoven increased with the charging temperature, and the highest surface voltage was obtained at 50 °C [14]. Kilic et al. charged the melt blown nonwoven at 130 °C and found that thermal charging resulted in a greatly enhanced filtration performance because the thermal conditions soften the polymer and enhance the mobility of dipolar molecules [15]. Annealing treatment can also improve the charge storage stability. Tabti et al. heated the material at 75 °C in an electric oven before charging. The results showed that the stability of the material surface voltage improved due to the reduction in the moisture content of the heated sample, and the charge decay slowed down as water increased the electrical conduction [16]. However, the studies involving the charging of materials at only one temperature have several shortcomings, and the optimal charging temperature requires further discussion. In addition, there are different opinions on which mechanism to use to improve filtration performance, and this should be further studied.

In this study, the polypropylene melt blown nonwovens were produced, and thermally stimulated charging methods, including annealing treatment before charging, heat charging, and cyclic charging methods, were adopted to improve their filtration performances. The web structure, filtration efficiency and pressure drop, filtration efficiency decay, surface voltage decay, and thermally stimulated discharge (TSD) current spectrum were measured to evaluate the filtration and electret performances. The mechanism for enhancing the filtration performance was further discussed.

## 2. Materials and Methods

### 2.1. Materials

Polypropylene (PP) polymer resin with a melt flow index of 38 g/10 min was purchased from Shanghai SECCO Petrochemical Company Limited (Shanghai, China).

### 2.2. Preparation of Melt Blown Nonwovens

**Figure 1:**
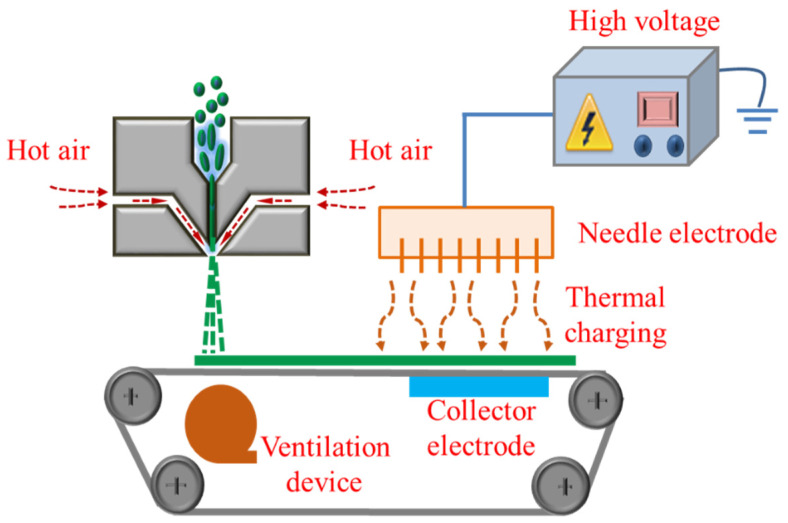
Schematic of the fabrication procedure of charged polypropylene (PP) melt blown nonwovens.

Melt blown nonwovens with a density of 40 ± 1 g/m^2^ were produced using a self-developed, laboratory scale melt blown machine. The diameter of the hole was 0.25 mm, and the air plate angle was 60°. The fabrication procedure of the charged PP melt blown nonwovens is presented in Figure 1, and the main melt blowing process parameters are listed in Table 1.

### 2.3. Corona Charging

The corona charging apparatus consisted of one high-voltage power supplier, one needle electrode, and one collector electrode (Figure 1). As previously mentioned, three parameters mainly influence the charging properties, namely, applied voltage, charging distance, and charging time. In this study, all melt blown nonwovens were charged at a voltage of 120 kV. The charging time and distance were 40 s and 10 cm, respectively. The thermal stimulus method was adopted during corona charging. The details are discussed in the following subsections.

#### 2.3.1. Annealing Treatment before Charging

The nonwovens were heated at 70, 100, and 140 °C for 2 h in an oven and subsequently cooled. The annealed melt blown nonwovens were then charged at room temperature (25 °C).

#### 2.3.2. Thermal Charging

The melt blown nonwovens were charged at 25, 70, 100, and 140 °C, by using a heating apparatus, and the temperature was controlled by adjusting the heating voltage.

#### 2.3.3. Cyclic Charging

During the cyclic charging process, the samples were charged at room temperature at first, then the samples were heated at the temperature of 140 °C in an oven for 10 min, after that the samples were charged again, this process was one cycle. After one cycle, the material went through a process of heat treatment and charging again, this process was called cycle 2. The number of cycles of different samples were 0, 1, 2, and 3.

### 2.4. Characterization of the Melt Blown Nonwovens

The surface morphology of the melt blown nonwovens was investigated using a scanning electron microscope (SEM; DXS-10ACKT, Shanghai Tianjing Electronic Optics Co., Ltd., Shanghai, China). The fiber diameter and distribution were calculated using the software Nano Measurer 1.2.5 by measuring 200 fibers from the SEM image. The pore structure of the melt blown nonwovens was measured using a capillary flow porometer (CFP-1100AI, Porous Materials Inc., Ithaca, NY, USA) based on the bubble point test. During the measurement, the pores of the sample were first filled with a wetting liquid, then the liquid was emptied by a nonreacting pressurized gas. After all the liquid was extruded from the pores, gas blew through the same dry sample. The differential pressures and gas flow rates through wet and dry samples were measured. The pore size (maximum, minimum pore diameter, and mean flow pore diameter) and distribution were calculated by the software from Quantachrome Instruments. The mechanical properties were tested via a tensile tester (YG026MB, Wenzhou Fangyuan Instrument Co., Ltd., Wenzhou, China). The length, width, and thickness of the tested samples were 200, 50, and 0.40 mm, respectively. The speed of the mechanical tester was 100 mm/min. The annealing treatment experiments were performed in a chamber (H/GDWJS-50L, Shanghai Husheng Instruments Co. Ltd., Shanghai, China) to treat the sample at different temperatures for 24 h.

The filtration performances of the electret melt blown nonwovens were tested using an automated filter machine (TSI8130, TSI Instruments Co., Ltd., Shoreview, MN, USA). The aqueous NaCl solution with a concentration of 2% was used to generate the particles by the aerosol generator. The mass median diameter of aerosol was 0.26 μm, and the count medium was 0.075 μm. An electron-laser particle photometer was used to measure the concentration of the aerosol particles in the upstream (Cu) and downstream (Cd), respectively. Filtration efficiency η was calculated as follows:

(1) $$\eta =(1-\frac{c_{d}}{c_{u}})\times 100\%$$

Each sample was tested five times to ensure accuracy. The pressure drop of the sample was measured by a flow gauge and two electronical pressure transmitters. The test process was carried out at an ambient temperature of 25 ± 2 °C and a relative humidity of 45 ± 5%. The air velocity was 14.1 cm/s. The surface electrostatic potential was measured using a non-contacting voltmeter (Trek 542-A, TREK Inc., Lockport, NY, USA). Three different points were tested for each sample to ensure accuracy. The electret properties were investigated through TSD techniques. The open-circuit TSD measurements were conducted in a system consisting of a temperature-controlled oven with a linear heating rate of 3 °C/min, an electrometer (Keithley Model 6514, Beaverton, OR, USA), and a data processing computer. The wide-angle X-ray diffraction (XRD) was analyzed using an X-ray diffract meter (D/max-2550 PC, Tokyo, Japan) in continuous mode to calculate the degree of crystallinity and crystal structure.

## 3. Results

### 3.1. Melt Blown Nonwoven Web Properties

**Figure 2:**
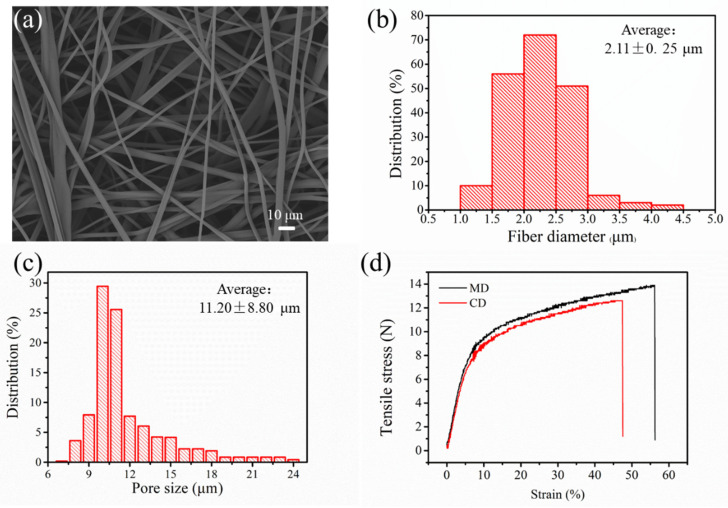
Web properties of melt blown nonwovens: (**a**) SEM image, (**b**) fiber diameter distribution, (**c**) pore size distribution, and (**d**) tensile strength.

Figure 2 presents the web structure of the as-prepared PP melt blown nonwovens. The scanning electron microscope (SEM) image (Figure 2a) reveals that a 3D micro porous structure (porosity ~ 90%) was formed by the randomly oriented fibers. The fibers have an average diameter of 2.11 ± 0.25 μm (Figure 2b), and the average pore size is 11.20 ± 8.80 μm (Figure 2c). On the one hand, the 3D micro porous structure can serve as an effective channel for the traverse of air molecules, and the small pore size can facilitate the mechanical filtration for particles based on the mechanism of physical intercept, diffusion, and inertial impaction [17]. On the other hand, the fine particles can also be captured by the electrostatic attraction effects after charging. The results indicate that the electret melt blown nonwoven is a good candidate for an air filter with high efficiency and low air resistance. The mechanical property is a noteworthy factor that can determine the practical applications of this material. As shown in Figure 2d, the machine direction (MD) demonstrated a higher tensile stress and larger strain than the cross-machine direction (CD), which might be due to the arrangement of more fibers along the output direction of the machine despite the random distribution of the fibers. In addition, the breaking strength of the CD was 12 N, which was sufficiently high for most air filtration applications, was because the melt blown nonwoven was not used as a filter material alone and often combined with other materials. For example, melt blown was used as the core filter layer of mask, and the spunbond nonwoven mainly provided the mechanical strength.

### 3.2. Filtration Performances of Samples That Are Annealed before Charging

**Figure 3:**
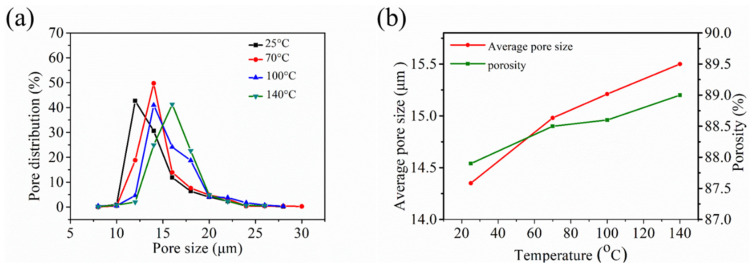
Pore structure of the annealed melt blown samples: (**a**) pore distribution, and (**b**) average pore size and porosity.

The pore structure plays a major role in the traversal of air molecules, and thus affects the air resistance. Figure 3a shows that the pore distribution displayed a similar behavior with the increase in annealing temperature, except for the peak that migrates to an increased pore size. Moreover, the average pore size and porosity increased after the annealing treatment (Figure 3b). This phenomenon can be ascribed to the expansion of the pores due to the heat treatment, which resulted in the fluffy structure of the melt blown nonwoven. This result shows that the air resistance will also decrease after the annealing treatment.

**Figure 4:**
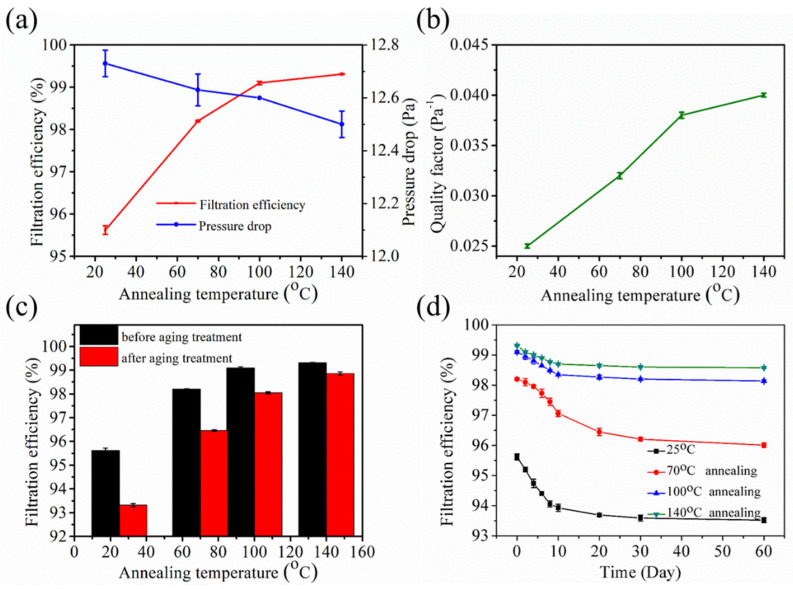
Filtration performances of the annealed melt blown samples: (**a**) filtration efficiency and pressure drop, (**b**) quality factor, (**c**) filtration efficiency decay after the aging treatment, and (**d**) filtration efficiency decay after two months.

The filtration performances of the annealed samples are shown in Figure 4. The filtration efficiency of the non-annealed sample was 95.62%, whereas those of the samples annealed at 70, 100, and 140 °C were 98.20, 99.10, and 99.31%, respectively (Figure 4a). The filtration efficiency exhibits an escalating tendency with the increase in the annealing temperature. Meanwhile, the pressure drop showed a slight decreasing trend, which can be attributed to the large pore size and high porosity caused by the expansive structure that allowed the air molecules to easily pass through the aperture channels. The quality factor (*QF*), which acts as an indicator that can comprehensively evaluate the filter performances, was calculated as.

(2) $$QF=(ln\frac{1}{1-\eta })/∆p$$

where *η* is the filtration efficiency and ∆*p* represents the pressure drop [18]. The behavior of *QF* was similar to that of the filtration efficiency (Figure 4b). This finding indicates that the annealing treatment can improve the comprehensive filtration performance, and a high annealing temperature results in a high *QF*. As the annealing temperature increased, the filtration efficiency increased, whereas the pressure drop declined; this condition is conducive for improving the *QF*.

The stability of the filtration efficiency is a great property of electret filtration materials, especially because charge attenuation will lead to a decrease in the filtration efficiency. Therefore, aging and long-term filtration decay experiments were performed. The filtration efficiency of the non-annealed sample after the aging treatment was 2.30%, whereas those of the samples annealed at 70, 100, and 140 °C were 1.74, 1.05, and 0.45%, respectively (Figure 4c). This result implies that the aging resistance stability of the electret materials is improved by performing annealing treatment before charging. Figure 4d shows that the filtration efficiency exhibited a gradually decreasing trend versus the storage time. After two months, the filtration efficiency reached an almost constant value, and the decrements in the efficiency of the non-annealed sample and the samples annealed at 70, 100, and 140 °C were 2.10, 2.09, 0.96, and 0.73%, respectively. This finding indicated that the annealing treatment can also improve the stability of the long-term filtration efficiency, especially that of samples annealed at high temperature. Furthermore, the surface voltage attenuation was non-linear as it rapidly decayed in the first month and slowly declined in the second month.

**Figure 5:**
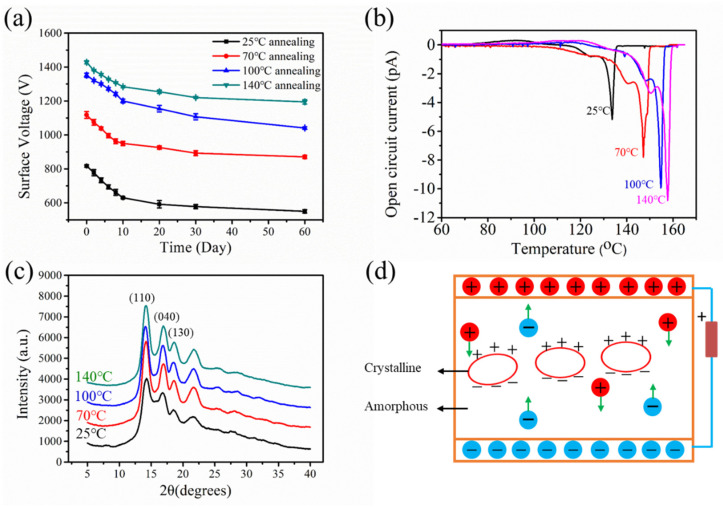
Electret properties of the annealed melt blown samples: (**a**) surface voltage decay after two months, (**b**) thermally stimulated discharge (TSD) spectra, (**c**) X-ray diffraction (XRD) pattern, and (**d**) schematic of piled charges at the crystalline surface.

To reveal the mechanism of the filtration efficiency attenuation, surface potential decay experiments were conducted. As illustrated in Figure 5a, the surface voltage displayed a similar decay trend as the filtration efficiency, thereby confirming that the decay of the former was the main reason of the decrease in filtration efficiency. The attenuations of the surface voltage after two months were 367, 287, 240, and 233 V; such values suggest that the annealing treatment can improve the charge storage stability. The finding further reveals that the surface voltage decay curve can be divided into two stages. In the first month, the curve was quite steep, and the voltage drastically declined before becoming flat and decreasing in a slow manner. This phenomenon can be explained by the existence of two kinds of charges in the electret material, namely, the quick-decay and slow-decay charges, which respectively correspond to the shallow and deep charges [19,20]. Given that the shallow charges on the material surface easily escaped, the voltage rapidly decayed at first. The slow-decay charges were stored in the deep traps and released in a slow process; such phenomena caused the curve to flatten and the voltage to slowly decline after one month.

To further uncover the mechanism of the surface voltage storage stability, the electret properties of the samples were investigated through TSD measurement. The TSD current measurement is widely used to investigate the charge trap factors in polymers due to the high sensitivity of the charge trap parameters to the structure of the polymers [21]. Figure 5b depicts that the shapes of the peaks were similar, whereas the peak intensities and the corresponding temperatures were different from each other. Two peaks appeared for all samples, which confirmed that two kinds of charges were present in the material. The shoulder peak can be attributed to the release of the shallow trap charge, whereas the main peak was due to the escape of the deep trap charge [22]. The shoulder and main peaks of the non-annealed sample appeared at 125 and 133 °C, respectively. The shoulder peaks of the samples annealed at 70, 100, and 140 °C were respectively observed at approximately 140, 148, and 150 °C, whereas the corresponding main peaks appeared at 147, 155, and 158 °C, respectively. The significant shift of the peak temperature to high temperature indicated that the trap depth of the annealed samples deepened, and the de-trapping phenomenon was limited under thermal stimulation condition [23]. A gentle current appeared at 75 °C for the non-annealed sample and at 100, 119, and 122 °C for the samples annealed at 70, 100, and 140 °C, respectively. The escape of the charges at high temperature further confirmed that the charge storage stability improved. Moreover, the current peak intensity and peak area increased, which means that additional charges were captured by the traps. This phenomenon can be explained by the fact that the crystal structure changed during the annealing process, and new charge traps were created. The crystal structures were investigated, and the crystal parameters were calculated (Figure 5c and Table 2). The lattice planes (110) appeared at 13.90°, 14.00°, 14.05°, and 14.10° for the non-annealed sample and the samples annealed at three different temperatures, respectively; such an appearance indicated that the materials were predominately crystallized into α-crystals [24]. The crystallinity increased from 40.91 to 58.35% after the sample was annealed at 140 °C, and the interplanar spacing and spherulites size decreased after the annealing treatment (Table 2). This result proved that the annealing treatment can change the crystal structures. Previous studies stated that the charge traps mainly existed in the interfaces among crystallites or between the crystallite and the amorphous region of the PP materials [25]. Owing to the different electrical conductivity between the crystallite and the amorphous region, the motion of the charge carriers was limited, and these carriers were deposited on the surface of the former [26] (Figure 5d). Furthermore, the shallow traps in the PP exist in the boundaries and peripheral regions of the spherulites, whereas the deep ones are located in the central parts. The ratio of deep traps to shallow traps increases with the decrease in spherulites size [22]. Consequently, the annealing treatment created an optimized crystal structure formed by the deep traps, and thus improved the electret performance.

### 3.3. Filtration Performances of the Thermally Charged Samples

**Figure 6:**
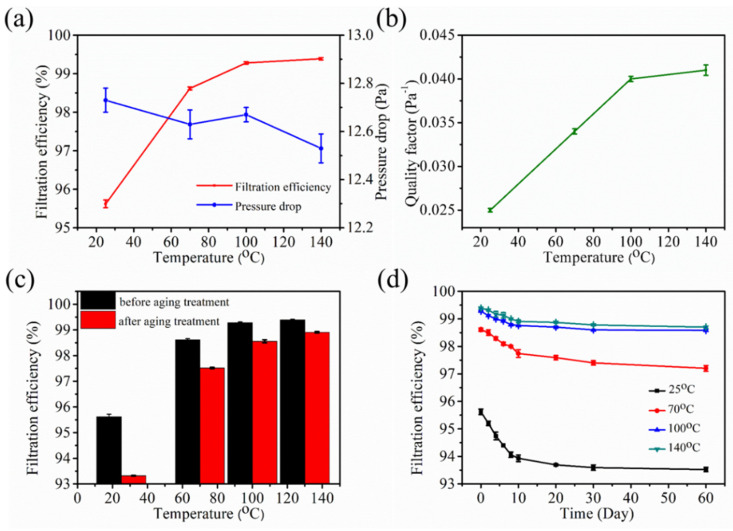
Filtration performances of thermally charged melt blown samples: (**a**) filtration efficiency and pressure drop, (**b**) quality factor (*QF*), (**c**) filtration efficiency decay after the aging treatment, and (**d**) filtration efficiency decay after two months.

The PP melt blown nonwovens were charged at elevated temperatures. The results showed that the filtration efficiency and *QF* improved with the increase in charging temperature (Figure 6a,b). The sample charged at 140 °C achieved a filtration efficiency of 99.41%, thereby surpassing the sample charged at 25 °C (95.62%). This phenomenon can be ascribed to the superior electret performances of the samples charged at an elevated temperature, as well as to the increased number of particles captured by the electrostatic attraction mechanism.

The stability of the filtration efficiency was subsequently investigated. As illustrated in Figure 6c, the filtration efficiencies of the samples charged at 25, 70, 100, and 140 °C after the aging treatment decreased by 2.30, 1.10, 0.72, and 0.48%, respectively. These decrements indicated that the aging resistance stability improved, which might have been due to the movement of the charges deposited at the surface of the material to deeper traps at high temperature. These charges were relatively stable under aging condition [27]. In addition, the long-term stability of the filtration efficiency was measured. The filtration efficiencies of the four charged samples declined after two months by 2.10, 1.41, 0.70, and 0.60%, respectively (Figure 6d). This finding revealed that the thermal charging technology significantly enhanced the long-term attenuation property of the filtration efficiency.

**Figure 7:**
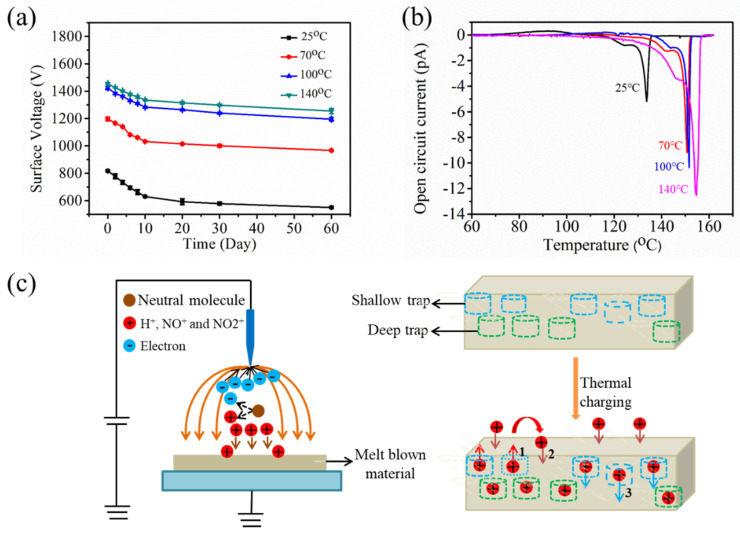
Electret properties of melt blown samples with thermal charging: (**a**) surface voltage decay after two months, (**b**) TSD spectra, and (**c**) schematic of piled charges during the thermal charging process.

The surface voltage displayed a similar decay tendency as the filtration efficiency (Figure 7a), which means that the thermal charging technology can improve the stability of the electret performances. The electret properties were further analyzed using the TSD method (Figure 7b). The shoulder and main peaks of the samples charged at 25, 70, 100, and 140 °C appeared at 125 and 133, 142 and 151, 144 and 152, and 146 and 155 °C, respectively. Both current peaks shifted to high temperature and therefore demonstrated an obvious change in the charge stability. This shift can be attributed to a re-trapping phenomenon, in which the de-trapped charges undergo drift and a second deep-trapping process at high temperature, and the mean charge depth remarkably deepens. Figure 7c displays the model of charge distribution and drift in a thermal positively charged process. Corona discharge occured due to the high potential difference between the high voltage source and the receiving electrode. This phenomenon led to the breakdown of the neutral molecule and caused the ions to move toward the low-potential electrode. In this study, we utilized a positive power to allow the electric field to ionize the neutral molecules to generate H+, NO+, and NO2+ ions [28]. The ions drifted toward the insulator and be deposited on the surface and transferred electrons to the traps. The charges injected into the traps under electric field. Meanwhile, several charges were released from the traps via thermal activation and drift in their own fields (arrow 1 in Figure 7c). These charges were then be re-trapped again after a mean free path in energetically deep trap centers, which were located deep in the bulk of the sample (arrow 2 in Figure 7c). In addition, the activated de-trapped charge might drift downward to the rear contact electrode due to thermal stimulation (arrow 3 in Figure 7c); such a drift will greatly increase the charge trap depth. Consequently, the trap energy bands changed to high bands, and the charge storage stability improved. Furthermore, the current peak intensity and peak area of de-trapped charge at high temperatures were larger than those at low temperatures. This discrepancy indicates that most of the implanted charges were in deep traps and thermally stable.

### 3.4. Filtration Performances of the Samples with Cyclic Corona Charging

An innovative charging technology, which is called cyclic charging, was adopted to further improve the electret performances. The detailed procedure of cyclic charging is presented Section 2.3.3. The samples were heated at 140 °C because the charges start to escape from the TSD spectra at this temperature. The cycle numbers for different samples were 0, 1, 2, and 3, respectively.

**Figure 8:**
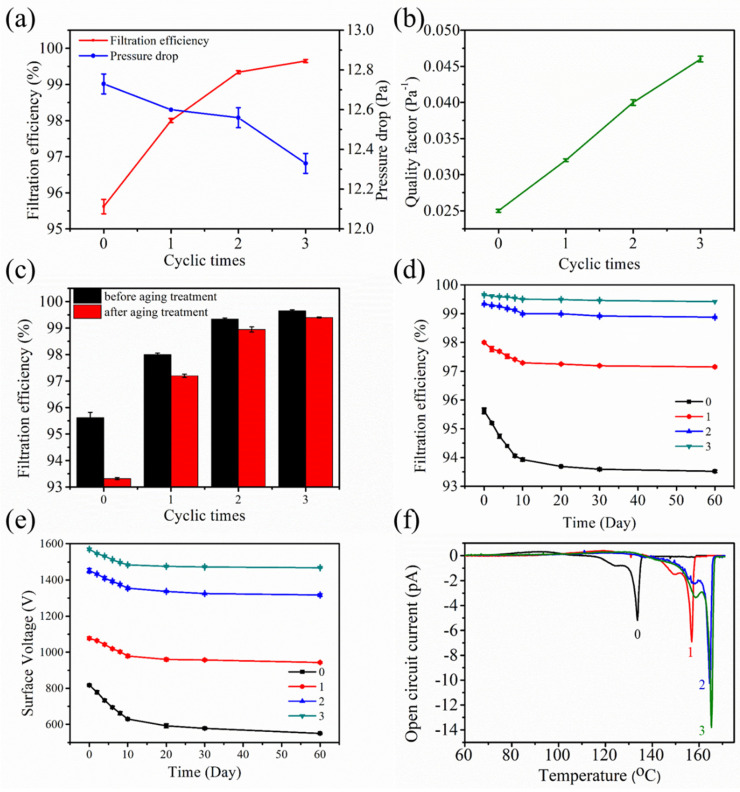
Filtration and electret performances of the samples with cyclic corona charging: (**a**) filtration efficiency and pressure drop, (**b**) *QF*, (**c**) filtration efficiency after the aging treatment, (**d**) filtration efficiency decay after two months, (**e**) surface voltage decay after two months, (**f**) open-circuit TSD current.

The filtration performances of the electret melt blown nonwovens that undergo a cyclic corona charging process were monitored. As shown in Figure 8a,b, the filtration efficiency and *QF* increase as the cyclic charging time increased. The filtration efficiency after three cyclic charging was 99.65%. This result indicated that cyclic charging can improve the filtration performance; such an improvement can be ascribed to the enhancement of the surface voltage. The filtration efficiencies of the samples after the aging treatment were 2.30, 0.80, 0.49, and 0.25%, respectively (Figure 8c). Meanwhile, the long-term filtration efficiency attenuations after two months were 2.10, 0.85, 0.46, and 0.23%, respectively (Figure 8d). These results imply that the aging resistance and long-term stability of the filtration efficiency were enhanced by the cyclic charging technology; the decay of the surface voltage further confirmed this inference (Figure 8e). We confirmed that the filtration efficiency would reach a stable value when the charging time exceeded the threshold value because the traps were saturated with the increase in charging time [29]. We set the charging time to 40 s and excluded the possibility that the improvement in the filtration efficiency was due to the indirect increase in charging time. We assumed that the cyclic corona charging method changes the charge distribution in the electret material. The deep and shallow charges might de-trap from the traps due to thermal activation after heat treatment. However, the shallow charges trapped at the low band easily escaped, whereas the deep charges stored at the high band were relatively stable [20]. The activated charges also drift downward to the rear contact electrode to store in the deep level. Subsequently, the newly ionized charges caused by the recharging process were implanted to the material and captured by the empty traps. The cyclic charging method released some of the charges from the shallow traps and then deeply trapped them again. As a result, numerous shallow charges transformed into deep charges, which greatly improved the proportion of deep charge, as well as the charge stability.

The electret properties were further investigated using the TSD current spectra. Comparing spectrum 0 with spectrum 3, the main peak temperature shifted from 133 to 165 °C after three cycles, and thus indicated that the energy level was effectively deepened (Figure 8f). In addition, the intensity and peak area of the main peak in the latter are higher than those in the former. This finding was consistent with the initial surface potential of the samples and therefore confirm that the cyclic corona charging process was beneficial to the improvement of the electret performance due to the change of the trap energy bands to high bands. In addition, this charging process was related to the local change in the charge center from the near surface region into bulk [30].

## 4. Conclusions

COVID-19 is an exceptionally contagious disease that requires the public to take many precautions including the use of N95 respirators to protect themselves. In this study, we designed a PP electret melt blown nonwovens for N95 respirators with high filtration and superior filtration efficiency stability. Thermally stimulated charging method, such as annealing treatment before charging, thermal charging, and cycle charging were adopted during the corona charging process. The quantity and depth of the charge traps greatly improved due to the optimized crystal structure and thermal stimulation. A superior electret melt blown filtration material with a high filtration efficiency of 99.65%, a low pressure drop of 120 Pa, and a satisfactory stability was produced after three cyclic charging times at an air velocity of 14.1 cm/s and particles with a mass median diameter of 0.26 μm. This value was far superior to the current personal respirator standard. The results indicated that the material would provide guidance to the field of air filtration, especially in COVID-19 virus protection applications.

## Figures and Tables

**Table 1:** Melt blowing process parameters.

Parameters	Screw 1 Temperature (°C)	Screw 2 Temperature (°C)	Screw 3 Temperature (°C)	Air Temperature (°C)	Distance (mm)	Air Pressure (MPa)
	280	325	330	300	100	0.20

**Table 2:** Crystallinity of the samples with annealing treatment.

Sample	Crystallinity (%)	2θ(°)	D (Å)	XS (Å)
25 °C	40.91	13.90 16.90	6.256 5.233	115 132
70 °C annealing	44.98	14.00 16.90	6.281 5.227	103 122
100 °C annealing	49.24	14.05 17.10	6.232 5.202	98 115
140 °C annealing	58.35	14.10 17.15	6.215 5.185	83 103
